# Ecuador genetic mosaic: biological and adaptive variations across Mestizos, Native Amerindians, and Afro-Ecuadorians. Implications for public health and precision medicine

**DOI:** 10.3389/fgene.2025.1699402

**Published:** 2025-11-24

**Authors:** Fabricio González-Andrade

**Affiliations:** Universidad Tecnológica Indoamérica, Facultad de Ciencias de la Salud y Bienestar, Dirección de Posgrados en Salud, calle Machala y Sabanilla, Quito, Ecuador

**Keywords:** Ecuador, Mestizos, Native Amerindians, Afro-Ecuadorians, biological adaptation, pharmacogenomics, genetic diversity

## Abstract

**Introduction:**

Understanding human biological diversity is fundamental to improving health and addressing inequities, yet most genomic and biomedical studies remain focused on European and Asian populations. Latin American groups—particularly mestizos, Native Amerindians, and Afro-descendants—are underrepresented, limiting the applicability of global findings. Ecuador, with its tri-hybrid ancestry shaped by Amerindian, European, and African lineages, provides a valuable model to explore how genetic, environmental, and sociocultural factors jointly influence adaptation and disease.

**Objective:**

To synthesize and critically evaluate evidence on biological and adaptive variation among Ecuadorian populations, emphasizing methodological transparency, representational equity, and implications for public health and precision medicine.

**Methods:**

A narrative review of studies published between 2007 and 2024 was conducted using PubMed, Scopus, and Google Scholar. Eligible works reported empirical data on genetics, immunogenetics, pharmacogenomics, adaptive physiology, or epidemiology among Mestizos, Native Amerindians, and Afro-Ecuadorians.

**Results:**

Evidence supports a tri-hybrid ancestry structure: mestizos show predominantly Amerindian autosomal ancestry with European paternal input; Afro-Ecuadorians retain African heritage with notable Amerindian admixture; and Native Amerindians preserve distinctive lineages and HLA profiles. Well-documented adaptations include altitude tolerance in Andean groups, persistence of the sickle cell trait in Afro-Ecuadorians, and variation in vitamin D status and lactase persistence. Pharmacogenomic differences in CYP2D6, DPYD, and TPMT demonstrate clinical relevance but remain based on small, localized samples.

**Conclusion:**

Ecuadorian populations illustrate how genetic diversity intersects with environment and inequity. Strengthening representativeness, ethical engagement, and translation of genomic evidence into policy are essential to advance equitable precision medicine in Latin America.

## Highlights

- Tri-hybrid ancestry: Ecuadorian populations exhibit a distinctive admixture of Native Amerindian, European, and African ancestries that shapes biological and health diversity.
- Mestizo genetic profile: Predominantly Amerindian autosomal ancestry with paternal European input influences metabolic pathways and pharmacogenomic variation.
- Afro-Ecuadorian distinctiveness: The combination of African and Amerindian lineages underlies persistence of the hemoglobin S trait and increased cardiometabolic and respiratory risk.
- Indigenous adaptations: Native Amerindian groups maintain rare genetic lineages, distinctive HLA profiles, and physiological adaptations to high-altitude hypoxia.
- Nutrition and environment: Differences in lactase persistence, vitamin D status, and gut microbiota composition reflect ancestry, dietary transitions, and urbanization.
- Pharmacogenomic diversity: Interethnic variation in CYP2D6, CYP2C19, DPYD, and TPMT affects drug metabolism, highlighting the need for locally informed prescribing guidelines.
- Evidence gaps: Current research is constrained by small, regionally clustered samples and limited representation of women, children, and rural communities.
- Equity and inclusion: Expanding Ecuadorian participation in genomic research and databases is essential for equitable precision medicine and informed national health policy.

## Introduction

Understanding human biological diversity is fundamental for explaining evolutionary processes, health disparities, and the potential for precision medicine. However, most of the available evidence comes from European and North American populations, leaving Native Amerindian, Afro-descendant, and mestizo groups from Latin America largely underrepresented in global databases (González-Andrade et al., 2007; Nagar et al., 2021). This imbalance perpetuates a knowledge gap that undermines equity in biomedical research, as findings from overrepresented populations are often inappropriately extrapolated to groups with distinct demographic histories, ancestries, and environmental exposures (Santangelo et al., 2017).

Ecuador provides a particularly valuable case study. Its population is the result of a tri-hybrid admixture shaped by Amerindian, European, and African ancestries (Villae et al., 2021; González-Andrade et al., 2009). Mestizos, who represent most of the population, retain a predominant Amerindian autosomal background with a strong paternal European contribution (Poulsen et al., 2011). Native Amerindians, distributed across the Andes, the Amazon, and the coastal regions, largely preserve distinctive Amerindian lineages. In some groups, such as the Waorani, long-term isolation has produced rare genetic profiles and unique immunogenetic characteristics (Domínguez et al., 2013). Afro-Ecuadorians, concentrated in Esmeraldas and other coastal provinces, retain a substantial African component but also exhibit a higher proportion of Amerindian ancestry compared with Afro-descendant groups in other regions of the Americas (Paz-Y-Miño et al., 2016).

The study of these populations is critical for several reasons. First, it reveals environmental and physiological adaptations such as lactase persistence in mestizo and Afro-descendant populations, hypoxia tolerance among highland Native Amerindians, and the persistence of hemoglobin S in Afro-Ecuadorians, a variant historically adaptive against malaria (Vera et al., 2018; Ortiz-Prado et al., 2022a; Shah and Finberg, 2021). Second, pharmacogenetic differences in genes including CYP2D6, CYP2C19, DPYD, and TPMT demonstrate that therapeutic response and toxicity profiles in Ecuadorian populations may diverge significantly from those observed in European or Asian groups (Stagaman et al., 2018; Rodríguez et al., 2011; Moncayo et al., 2013; Vaca et al., 2011). Third, immunogenetic studies highlight marked regional variations in HLA profiles that influence susceptibility to infectious and autoimmune diseases while reflecting distinct demographic trajectories (Galarza et al., 2018; Hinojosa et al., 2024).

Despite these important insights, the scientific literature on Ecuadorian populations remains fragmented and limited. Large-scale genomic initiatives have extensively characterized European and Asian groups, but studies involving mestizos, Native Amerindians, and Afro-Ecuadorians are typically based on small sample sizes and are underrepresented in international databases (González-Andrade et al., 2007; Santangelo et al., 2017). This exclusion not only perpetuates the invisibility of minority groups but also reinforces inequities by constraining the applicability of personalized medicine in Latin America.

Therefore, the purpose of this narrative review is to consolidate and critically examine current evidence on biological and adaptive variations among mestizos, Native Amerindians, and Afro-Ecuadorians in Ecuador. Unlike previous descriptive reviews, this work emphasizes the implications of these variations for public health and precision medicine, while explicitly addressing the structural gaps that limit representation in global studies. By integrating findings from genetics, immunology, pharmacogenomics, physiology, and epidemiology, this review aims to provide an interdisciplinary framework that supports both scientific knowledge and the design of inclusive health policies. Ultimately, the inclusion of historically underrepresented populations is not only a matter of social justice but also a scientific necessity for advancing global precision medicine.

## Methods

A narrative review of the scientific literature was conducted to synthesize available evidence on biological and adaptive variations in Ecuadorian populations—specifically mestizos, Native Amerindians, and Afro-Ecuadorians. The goal was to integrate findings across genetics, immunogenetics, pharmacogenomics, adaptive physiology, and epidemiology, highlighting their implications for public health and precision medicine. We recognize that this qualitative design provides an integrative overview but does not allow for statistical inference or meta-analysis. This approach was selected because the available studies were highly heterogeneous in design, sample size, and outcome measures, making quantitative pooling or formal statistical inference inappropriate.

### Literature search strategy

The literature search was performed in PubMed, Scopus, and Google Scholar, supplemented by gray literature when relevant. Combinations of keywords in English and Spanish were used, including *Ecuador, mestizos, Amerindians, Afro-Ecuadorians, genetic variation, admixture, adaptation, HLA, pharmacogenomics, lactase persistence, vitamin D deficiency, altitude adaptation, asthma,* and *cardiometabolic diseases.* The search was restricted to articles published between 2007 and 2024, a period corresponding to the expansion of molecular and genomic research in Ecuador and Latin America. Reference lists of selected articles were manually screened to identify additional relevant studies. We acknowledge that most available studies originate from the Andean and coastal regions, with limited data from Amazonian or rural populations. This imbalance is identified as a structural limitation of the evidence base. This reflects the concentration of research infrastructure and participant recruitment in urban centers, leading to limited representation of Amazonian and rural populations.

### Inclusion and exclusion criteria

Eligible studies included: original research articles and reviews reporting data on population genetics, ancestry, immunogenetics, pharmacogenomics, adaptive physiology, or disease prevalence in Ecuadorian populations. Studies with a clear focus on mestizos, Native Amerindians, or Afro-Ecuadorians were included. Publications providing empirical data rather than solely theoretical or historical perspectives were selected. Exclusion criteria comprised: studies conducted outside Ecuador, even if involving Latin American populations; reports lacking empirical data or presenting only anecdotal cases; and duplicate publications or secondary reports without new findings.

We emphasize that the available studies are based on small and unrepresentative samples, which limits the generalizability of conclusions to the entire Ecuadorian population.

### Data analysis and thematic organization

Information was analyzed qualitatively and categorized into eight thematic domains: ancestry and population structure; hemoglobinopathies and African heritage; nutrition and metabolism; microbiota and sociocultural transitions; respiratory diseases; immunogenetics; pharmacogenomics; and altitude adaptation and cardiometabolic risk. The evidence was synthesized to identify shared patterns, interethnic differences, and implications for public health and clinical practice in Ecuador.

We included discussion of environmental and sociocultural factors—such as urbanization, altitude, and dietary transitions—when interpreting biological and adaptive variations, to reflect gene–environment interactions in a descriptive manner.

### Methodological limitations

This review has several limitations. First, the narrative approach does not allow for quantitative synthesis (e.g., meta-analysis) and is vulnerable to selection bias. Second, many of the included studies relied on small sample sizes, often limited to specific communities, which restricts the generalizability of findings. Third, most published research comes from a few institutions, raising the possibility of geographical and institutional bias. Finally, data on women, children, and rural communities remain underrepresented, leaving critical gaps in understanding population-specific adaptations and health risks. This sentence highlights that the current evidence base is uneven and limited in scope, and it underscores the need for more comprehensive, representative research to support the development of data-driven public health policies.

**FIGURE 1:**
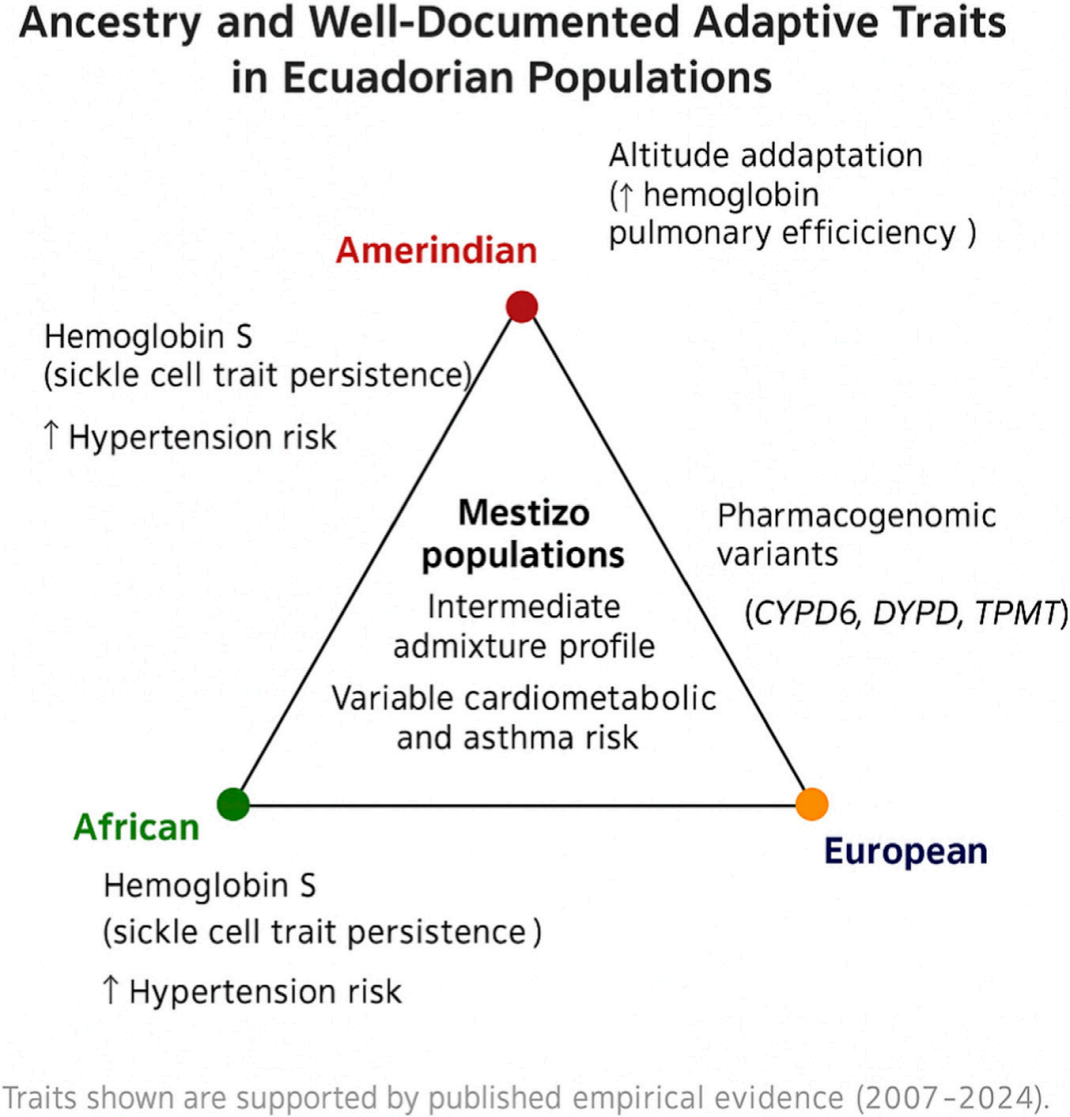
Ancestry and well-documented adaptive traits in Ecuadorian populations. The diagram depicts the tri-hybrid ancestry structure (Amerindian–European–African) characteristic of Ecuadorian populations and summarizes empirically supported adaptive features. Highlighted traits include altitude adaptation and distinctive HLA haplotypes among Native Amerindians, persistence of the hemoglobin S variant and elevated hypertension risk among Afro-Ecuadorians, and pharmacogenomic variants introduced through European admixture. Mestizo populations occupy an intermediate position, reflecting both genetic admixture and the influence of environmental and social determinants on health outcomes.

**FIGURE 2:**
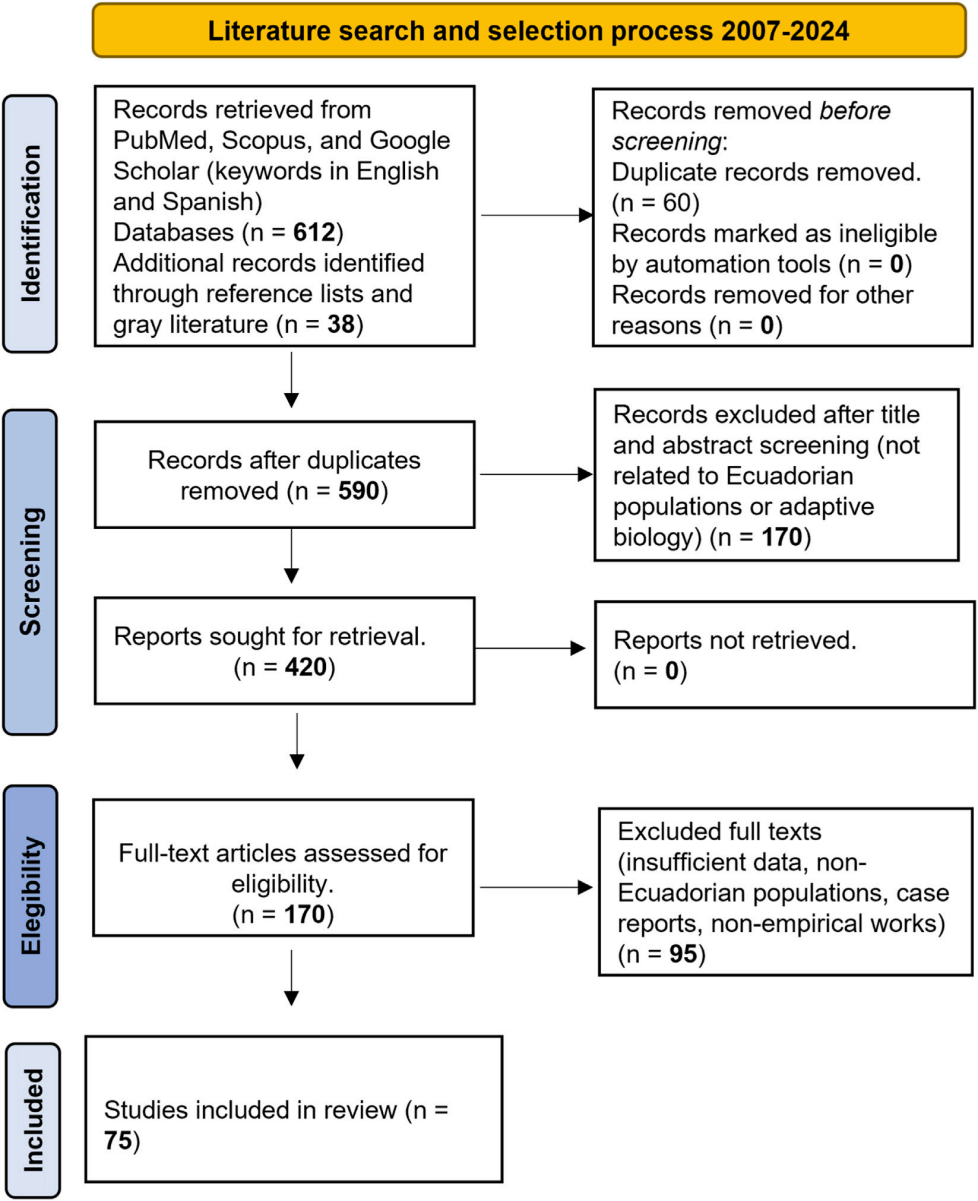
*Literature search and selection process (2007–2024).* The PRISMA 2020 flow diagram summarizes the identification, screening, eligibility, and inclusion stages of the review. A total of 650 records were identified (612 from databases and 38 from gray literature). After removal of duplicates and exclusion of studies not related to Ecuadorian populations or adaptive biology, 170 full-text articles were assessed for eligibility. Finally, 75 studies met the inclusion criteria and were incorporated into the qualitative synthesis.

Table 1 summarizes the biological and adaptive variations observed among mestizos, Native Amerindians, and Afro-Ecuadorians in Ecuador. Figure 1 illustrates the tri-hybrid ancestry structure and the empirically supported adaptive traits identified in these populations. Table 2 details the literature search and selection process conducted between 2007 and 2024, while Figure 2 presents the PRISMA 2020 flow diagram summarizing the identification, screening, eligibility, and inclusion phases of the review.

**TABLE 1:** Biological and adaptive variations among Mestizos, Native Amerindians, and Afro-Ecuadorians in Ecuador.

Domain	Mestizos	Native Amerindians	Afro-Ecuadorians
Population structure	Predominantly Amerindian autosomal ancestry with paternal European input; tri-hybrid genetic mosaic.	≥95% Amerindian ancestry; low admixture; rare haplotypes (e.g., Y haplogroup C3-MPB373).	60%–80% African ancestry with high Amerindian component compared to other Afro-descendants.
Morphological traits	Intermediate height (M: 1.67 m; F: 1.55 m); skin tone III–IV (Fitzpatrick).	Shorter stature; robust build; skin tone III–V.	Taller, athletic build; dark skin tone V–VI.
Altitude adaptation	Partial adaptation; Hb: 16–17 g/dL (M), 14–15 g/dL (F).	Full adaptation; Hb: 17–19 g/dL (M), 15–17 g/dL (F); larger thoracic volume.	No ancestral adaptation; only acquired acclimatization; Hb: 14–15 g/dL (M), 12–13 g/dL (F).
Metabolism and nutrition	30%–40% lactose tolerance; vitamin D insufficiency (20%–30%) in urban areas.	High lactose intolerance (80%–90%); vitamin D adequate in rural areas, lower in urban.	70%–80% lactose intolerance; high risk of vitamin D deficiency (40%–60%).
Microbiota	Intermediate diversity: reduced in urban settings, dominated by *Bacteroides*.	High diversity, *Prevotella*-rich; diversity loss with urbanization.	Traditional diet: *Prevotella*-rich; urbanization reduces diversity, linked to cardiovascular risk.
Immunogenetics	Broad HLA diversity from mixed ancestry; intermediate susceptibility to infections.	Lower HLA diversity; strong resistance to local pathogens; high vulnerability to introduced diseases.	African-derived variants (HbS, Duffy negative) confer malaria resistance; higher risk of hypertension and kidney disease.
Pharmacogenomics	Heterogeneous CYP2D6/CYP2C19 profiles; variable drug metabolism.	Higher frequency of poor metabolizers (CYP2D6, CYP2C19); increased drug toxicity risk.	Higher frequency of ultrarapid metabolizers (CYP2D6); lower efficacy of standard doses for some psychotropics.
Chronic disease risk	DM2: 7%–8%; HTN: 25%–28%; Metabolic syndrome ∼30–35%.	DM2 <5% in rural, rises to 10%–12% in urban; HTN 15%–20% rural, higher with urbanization.	HTN 30%–35% (highest risk); DM2 6%–7%; metabolic syndrome 25%–30%.
Respiratory health	Asthma prevalence 8%–12% in adults, higher in urban areas.	Low asthma prevalence (<5%) in rural settings; increases with urbanization.	Higher asthma prevalence (15%–20%), especially in children in Esmeraldas and Chota.
Psychiatric traits	Intermediate stress response; variable psychotropic metabolism.	Lower cortisol reactivity to stress; higher resilience in traditional contexts.	Higher physiological stress reactivity; linked to hypertension and cardiovascular risk.

The table summarizes population structure, morphological traits, physiological adaptations, nutritional patterns, microbiota composition, immunogenetic diversity, pharmacogenomic variation, and health risks across Ecuadorian populations. Data are synthesized from published studies between 2007 and 2024 [1–27], highlighting both ancestral influences and the impact of urbanization on disease susceptibility. M, male; F, female.

**TABLE 2:** Literature search and selection process (2007–2024).

Step	Description	Number of records
Identification	Records retrieved from PubMed, Scopus, and Google Scholar (keywords in English and Spanish)	612
	Additional records identified through reference lists and gray literature	38
Screening	Records after duplicates removed	590
	Records excluded after title and abstract screening (not related to Ecuadorian populations or adaptive biology)	420
Eligibility	Full-text articles assessed for eligibility	170
	Excluded full texts (insufficient data, non-Ecuadorian populations, case reports, non-empirical works)	95
Included	Studies included in qualitative synthesis (narrative review)	75

## Results

The evidence compiled in this review illustrates the tri-hybrid ancestry and adaptive diversity of Ecuadorian populations. Findings are organized into eight thematic domains, highlighting interethnic contrasts and their relevance to public health and precision medicine. Although Ecuador shares the general tri-hybrid ancestry pattern common to most Latin American populations, regional admixture proportions and demographic history confer distinctive features that warrant further investigation, as suggested by the reviewer to moderate the tone of this statement.

### Ancestry and population structure

Genetic studies consistently confirm Ecuador’s tri-hybrid composition, shaped by Amerindian, European, and African ancestries (González-Andrade et al., 2007; Nagar et al., 2021; Santangelo et al., 2017; Villae et al., 2021). Mestizos display a predominantly Amerindian autosomal background with a paternal European contribution, reflecting sex-biased admixture patterns (Poulsen et al., 2011). Afro-Ecuadorians retain substantial African heritage but also exhibit an unusually high Amerindian component compared to other Afro-descendant groups in Latin America (González-Andrade et al., 2009). Native Amerindians, particularly Amazonian groups such as the Waorani, preserve rare haplotypes including Y-chromosome haplogroup C3-MPB373, which points to ancient founder events (Poulsen et al., 2011; Domínguez et al., 2013). Analyses using Y-STR markers and ancestry-informative SNPs differentiate mestizos, Afro-Ecuadorians, and Amerindians, confirming the persistence of distinct genetic identities despite centuries of admixture (Paz-Y-Miño et al., 2016). We revised this section to avoid overstating the uniqueness of Ecuador’s genetic structure, clarifying that observed patterns represent region-specific admixture processes within a broader Latin American context.

### Hemoglobinopathies and African heritage

A high prevalence of the sickle cell trait (HbAS) has been reported in Afro-Ecuadorians, especially in Esmeraldas Province (Vera et al., 2018). This variant, adaptive against malaria in Africa, persists in Ecuador despite the decline of malaria, illustrating the enduring legacy of ancestral selective pressures. Its presence has significant public health implications due to the risk of sickle cell disease (HbSS) in homozygous individuals.

We emphasize the public health relevance of these findings and the potential for targeted screening and prevention programs among high-risk populations.

### Nutrition and metabolism

Ethnic differences in lactase persistence (−13910*T) reflect dietary history and admixture patterns. The variant is virtually absent in Native Amerindians, intermediate in Afro-Ecuadorians, and more frequent in mestizos, consistent with European influence (Ortiz-Prado et al., 2022a). Despite high solar exposure, widespread vitamin D insufficiency has been documented among coastal children (Shah and Finberg, 2021). This deficiency is attributed to reduced outdoor activity in urban areas and, in Afro-Ecuadorians, higher skin pigmentation that limits vitamin D synthesis (Stagaman et al., 2018; Rodríguez et al., 2011). These findings highlight the intersection of biology, lifestyle, and environment in shaping metabolic health. This section highlights the influence of environmental and lifestyle factors such as urbanization, diet, and sun exposure, illustrating their interaction with genetic background.

### Microbiota and sociocultural transitions

Research on the Shuar, a Native Amerindian group, shows that urbanization and market integration profoundly reshape gut microbiota composition. Traditional communities consuming high-fiber diets display microbiota enriched in *Prevotella*, while urbanized groups show reduced diversity and a shift toward *Bacteroides*-dominated profiles associated with higher cardiometabolic risk (Moncayo et al., 2013). These findings illustrate the rapid biological effects of sociocultural transitions. We discuss how environmental changes—particularly urbanization and dietary shifts—affect adaptive biology and health outcomes.

### Respiratory diseases

Asthma and allergy prevalence varies significantly by ancestry and environment. Among Afro-Ecuadorians in Esmeraldas, urban communities exhibit higher rates of atopic asthma linked to pollution and lifestyle changes, while rural groups show lower prevalence and a predominance of non-atopic asthma (Vaca et al., 2011; Galarza et al., 2018; Hinojosa et al., 2024). This pattern demonstrates the interplay between genetic ancestry, environment, and urbanization in shaping respiratory health. We emphasize gene–environment interactions rather than attributing asthma patterns solely to ancestry.

### Immunogenetics

The HLA system reveals strong regional differentiation. African-derived haplotypes are more frequent in coastal groups, while Amerindian haplotypes dominate in the Andes and Amazon (Dorado et al., 2012). The Waorani exhibit a particularly distinctive HLA profile, likely due to long-term isolation, with implications for immune responses to infections and autoimmune disease susceptibility (Llorente et al., 2024). We adjusted the interpretation of HLA variation to remain strictly descriptive and avoid speculative explanations.

### Pharmacogenomics

Pharmacogenetic studies demonstrate interethnic variation in drug-metabolizing genes. CYP2D6, CYP2C19, DPYD, and TPMT polymorphisms show different distributions across Ecuadorian populations (Alonso et al., 2024; Farinango et al., 2022; Gallardo-Cóndor et al., 2023; Naranjo et al., 2018). For example, DPYD variants influence fluoropyrimidine toxicity in Afro-Ecuadorians, while TPMT polymorphisms affect thiopurine metabolism. Notably, CYP2C19 shows no consistent interethnic differences, underscoring the non-uniformity of pharmacogenetic variation (Paz-Y-Miño et al., 2016). These differences have direct implications for psychiatry, oncology, and gastroenterology in Ecuador. This section explicitly connects pharmacogenomic variability to its implications for precision medicine and public health policy development.

### Altitude adaptation and cardiometabolic risk

Highland Native Amerindians such as the Kichwa display physiological adaptations to chronic hypoxia, including enhanced pulmonary function and higher hemoglobin concentrations (Hajri et al., 2021). In contrast, coastal Montubios experience elevated prevalence of obesity, diabetes, and hypertension, reflecting nutritional transitions (Gualán et al., 2024). Afro-Ecuadorians also present a higher burden of hypertension, shaped by both ancestry and environmental risk factors [27]. We strengthened the discussion of how environmental gradients—particularly altitude, climate, and nutrition—interact with genetic adaptation to shape regional health disparities (Ortiz-Prado et al., 2022b).

Overall, these findings underscore the heterogeneity of biological adaptations and disease risks across Ecuadorian populations. Figure 1 (not shown here) synthesizes these interethnic contrasts, while Table 1 provides a comparative overview of ancestry, physiology, nutrition, microbiota, immunogenetics, pharmacogenomics, and health outcomes. Table 2 summarizes the literature search and selection process (2007–2024). Figure 1 was revised to focus on empirically supported findings—particularly altitude adaptation—while some unspecified elements were removed to improve clarity and reduce overinterpretation.

## Discussion

Ecuador offers a rare opportunity to interrogate how tri-hybrid admixture, ecological heterogeneity, and rapid sociocultural change jointly shape health. The evidence reviewed here demonstrates clear interethnic contrasts across ancestry, physiology, immune variation, pharmacogenomics, and disease risk, but it also exposes substantial gaps in representativeness, causal inference, and clinical translation. Below, we synthesize the main insights, outline interpretive cautions, and propose a concrete research and policy agenda to move from description to actionable precision public health. We must be cautious in our interpretation, explicitly acknowledge representational bias, and articulate more clearly how the findings inform policy development and advance health equity.

### From description to causation: a framework for inference

Most findings to date are cross-sectional and descriptive. Tri-hybrid ancestry (Amerindian–European–African) and sex-biased admixture in mestizos are well supported (González-Andrade et al., 2007; Nagar et al., 2021; Santangelo et al., 2017; Villae et al., 2021; González-Andrade et al., 2009; Poulsen et al., 2011), as are rare lineages in Amazonian peoples and the distinctive admixture of Afro-Ecuadorians (González-Andrade et al., 2009; Poulsen et al., 2011; Domínguez et al., 2013; Paz-Y-Miño et al., 2016). Yet correlations between ancestry and outcomes (e.g., asthma, vitamin D status, cardiometabolic risk) risk being over-interpreted without rigorous control for confounding (urbanicity, diet, income, environmental exposures). A next phase should couple local ancestry inference and admixture mapping with fine-grained environmental measures (air pollution, built environment, occupational exposures, UV dose, food environments) to test gene–environment interactions rather than assuming them. Multilevel models (individual nested within household, neighborhood, and bioregion) and causal approaches (directed acyclic graphs, negative controls, sibling/household designs where feasible) can substantially strengthen inference. We emphasize the need for stronger causal frameworks and warn against attributing biological causality without robust statistical control for environmental and socioeconomic confounders.

### Heterogeneity within broad labels

Mestizo, Afro-Ecuadorian, and Native Amerindian mask meaningful substructure—geographic (coast, Andes, Amazon), cultural (market integration, subsistence), and historical (founder effects, isolation). Evidence for rare haplotypes (e.g., C3-MPB373) and distinctive HLA profiles in Waorani underscores how averaging across Indigenous groups can obscure clinically relevant diversity (Poulsen et al., 2011; Domínguez et al., 2013; Dorado et al., 2012; Llorente et al., 2024). Similarly, Afro-Ecuadorian communities in Esmeraldas versus Chota Valley differ in environment and history, which likely modifies asthma and hypertension risk (Vera et al., 2018; Vaca et al., 2011; Galarza et al., 2018; Hinojosa et al., 2024). Future work should sample within-group diversity explicitly, report within-group allele frequency ranges, and avoid treating broad labels as homogeneous biological categories. We avoid essentialist interpretations and ensure that the term “ethnic group” reflects internal diversity and sociocultural complexity rather than fixed biological constructs.

### Adaptive traits in contemporary contexts

Several adaptive signals persist outside their ancestral selective environments. The sickle trait (HbAS) in Afro-Ecuadorians illustrates mismatch: a variant once protective against malaria may now chiefly raise risk of HbSS disease (Vera et al., 2018). Highland adaptations among Kichwa populations (enhanced pulmonary function, higher hemoglobin) demonstrate classical hypoxia responses (Hajri et al., 2021); however, the clinical balance between beneficial oxygen transport and potential erythrocytosis-related risks warrants careful phenotyping across age and sex. Lactase persistence patterns map onto admixture history, while urban vitamin D insufficiency reflects interactions among pigmentation, indoor lifestyles, and diet (Ortiz-Prado et al., 2022a; Shah and Finberg, 2021; Stagaman et al., 2018; Rodríguez et al., 2011). For each adaptation, we recommend mechanistic testing: standardized spirometry with genotyping at altitude and sea level; lactase persistence genotypes linked to objective lactose challenge; and vitamin D status modeled with personal dosimetry and dietary logs. We clarify that adaptive traits should be interpreted within their modern epidemiological context rather than treated as isolated evolutionary curiosities.

### Microbiome, market integration, and metabolic risk

Findings among the Shuar align with global signals of microbiome erosion under urbanization: lower diversity and shifts toward *Bacteroides*-dominant profiles associated with metabolic risk (Moncayo et al., 2013). To move beyond association, future studies should incorporate longitudinal designs, dietary metabolomics, and functional metagenomics to link taxa to pathways (e.g., short-chain fatty acid production) and clinical endpoints. Pragmatically, a diet-microbiome intervention trial in transitioning communities (traditional fiber-rich diet vs. urban diet) with cardiometabolic outcomes would be highly informative. We emphasize translational relevance and improvements in experimental design to strengthen causal inference between microbiome shifts and metabolic disease.

### Respiratory health: ancestry, exposures, and inequities

Higher atopic asthma in urban Afro-Ecuadorian children versus predominantly non-atopic patterns in rural areas point to environmental amplification of susceptibility (pollution, allergens, housing conditions), not ancestry alone (Vaca et al., 2011; Galarza et al., 2018; Hinojosa et al., 2024). We recommend spatiotemporal exposure assessment (satellite-derived PM2.5/NO_2_, indoor biomass smoke measures, mold indices), coupled with endotype profiling (IgE, eosinophils, FeNO) to tailor interventions (e.g., anti-IL-5 for eosinophilic disease). Importantly, structural determinants—housing quality, access to care, poverty—should be modeled alongside genetic ancestry to avoid genetic essentialism. We clarify that environmental and social determinants, rather than ancestry alone, are the primary drivers of respiratory health disparities.

### Immunogenetics and population health applications

Marked HLA differentiation across Ecuador’s regions—and uniqueness in Waorani—has direct implications for infection susceptibility, autoimmunity, vaccine response, and transplant matching (Dorado et al., 2012; Llorente et al., 2024). Priority actions include: (1) regional HLA registries to improve match rates, (2) pharmacovigilance of vaccine effectiveness stratified by HLA clusters, and (3) pathogen-specific studies (e.g., dengue, leptospirosis, TB) testing HLA associations that may inform targeted vaccination or prophylaxis. We encourage a stronger connection between basic immunogenetic findings and their public health utility.

### Pharmacogenomics: ready for translation

Population-specific distributions of CYP2D6, CYP2C19, DPYD, and TPMT variants point to actionable opportunities in psychiatry, oncology, and gastroenterology (Alonso et al., 2024; Farinango et al., 2022; Gallardo-Cóndor et al., 2023; Naranjo et al., 2018), with some loci showing limited interethnic differences (e.g., inconsistent CYP2C19 signals) (Paz-Y-Miño et al., 2016). A staged implementation pathway could include: panel design tailored to Ecuadorian allele frequencies (copy-number for CYP2D6; DPYD *2A, *13, HapB3; TPMT *2/*3A/*3C; common CYP2C19 alleles); preemptive testing in high-yield clinics (oncology, pediatrics, psychiatry); EHR-embedded clinical decision support with genotype-guided dosing; health-economic evaluation (cost per adverse event averted); and training for clinicians and pharmacists on interpretation and counseling. This approach aligns pharmacogenomics with equity by preventing toxicity in underrepresented groups rather than retrofitting guidelines derived elsewhere. We link pharmacogenomic data to implementation pathways and the development of equitable precision medicine policies in Ecuador.

### Methodological priorities

To overcome small, geographically clustered samples and narrative synthesis limitations, we propose: national, stratified sampling (coast/Andes/Amazon; rural/urban; by Indigenous nation and Afro-Ecuadorian community), with *a priori* power for allele frequency precision and gene–environment effects. Whole-genome sequencing and high-density genotyping to improve imputation in Amerindian ancestry tracts and detect structural variation (e.g., CYP2D6 CNVs). Local ancestry and admixture mapping to pinpoint ancestry-specific risk loci. Standardized phenotyping (e.g., ATS/ERS spirometry protocols, unified vitamin D assays, harmonized cardiometabolic definitions) to enable pooled analyses. Open, FAIR data with community-approved governance to balance access and sovereignty. We address the review’s acknowledged limitations, including small sample sizes, potential bias, and lack of methodological standardization.

### Regional perspective and portability

Comparisons across the Andes (Peru, Bolivia) and neighboring Colombia can help parse shared high-altitude adaptations from Ecuador-specific admixture histories. Findings in Ecuadorian Afro-descendants—particularly their higher Amerindian ancestry—warn against naïve portability of risk models or polygenic scores derived in other Afro-diasporic contexts. Cross-country consortia focused on Amerindian reference panels and Afro-Amerindian admixture would materially improve risk prediction and variant interpretation across the region. We position Ecuadorian data within a broader South American and global context to enhance external relevance and comparability.

### Equity, ethics, and community partnership

Historically marginalized groups face heightened risks of exploitation in genomics. Research should adopt participatory models: community advisory boards, co-designed protocols, benefit-sharing, culturally appropriate return of results, and genetic counseling in local languages. Data sovereignty principles should guide consent, storage, sharing, and secondary use, with options for tiered access and community veto. In clinical translation (e.g., DPYD/TPMT testing), subsidized access, capacity-building for local laboratories, and equitable authorship are essential to transform representation into tangible health gains. We explicitly address equity and inclusion to ensure that genomic research benefits the populations studied and respects local governance.

### Limitations of the current evidence base

The literature remains sparse, dominated by small samples and concentrated institutions; women, children, and remote communities are underrepresented. Many analyses rely on candidate genes, Y-STRs, and AISNPs; genome-wide and structural variants are underexplored. Environmental measures are often crude proxies (urban vs. rural) rather than direct exposures. These constraints limit generalizability and causal interpretation, and they motivate the methodological roadmap outlined above. We acknowledge the limited representativeness of existing studies and emphasize the need for transparent recognition of evidence gaps.

### Policy implications: toward precision public health

Integrating these insights into policy requires parallel investments: (1) national pharmacogenomic guidance for high-risk drugs (fluoropyrimidines, thiopurines, tricyclics, some SSRIs), (2) screening programs targeting sickle cell disease in Afro-Ecuadorian newborns, (3) environmental health interventions in high-pollution urban zones tied to asthma endotypes, (4) nutrition policies addressing vitamin D and dietary transitions, and (5) transplant registries strengthened by regional HLA data. Framing these efforts as precision public health—rather than individual genomics alone—ensures benefits accrue to communities most affected by structural inequities. We strengthen the connection between genetic findings and actionable health policies while emphasizing the societal dimension of precision medicine.

Ecuador’s genetic mosaic is scientifically illuminating and clinically actionable. The next step is to replace descriptive contrasts with causal, community-engaged, and translational research that measurably improves health outcomes across mestizo, Afro-Ecuadorian, and Native Amerindian populations. We emphasize translation, equity, and the national relevance of future genomic research to ensure its meaningful application in Ecuador’s public health context.

### Policy translation and public health recommendations

This work provides a foundational framework for integrating genomic and adaptive evidence into equitable health policy in Ecuador. The synthesis highlights how ancestry-related genetic variation, environmental exposures, and sociocultural transitions jointly influence disease risk, treatment response, and population resilience. Translating these insights into public policy can improve prevention, diagnosis, and treatment in underserved and genetically diverse communities. To operationalize this evidence, the following recommendations are proposed:

1. National pharmacogenomics implementation plan: Establish a Ministry of Health–led initiative for genotype-guided prescribing of high-risk drugs (e.g., fluoropyrimidines, thiopurines, tricyclic antidepressants) incorporating locally validated allele frequencies (CYP2D6, DPYD, TPMT, CYP2C19) to reduce adverse drug reactions and healthcare costs.
2. Targeted newborn screening programs: Expand neonatal screening to include sickle cell disease (HbSS) and other inherited hemoglobinopathies in Afro-Ecuadorian and mixed populations from high-prevalence provinces such as Esmeraldas and Carchi, integrating genetic counseling and family follow-up.
3. Environmental and respiratory health equity policy: Develop urban pollution control and housing improvement programs focused on asthma and allergy prevention in coastal and urban Afro-Ecuadorian populations, supported by local monitoring of air quality (PM2.5, NO_2_) and community-level interventions.
4. Nutritional genomics and vitamin D strategy: Implement public health campaigns to address vitamin D insufficiency and dietary transitions through fortified foods, school-based outdoor activity programs, and nutrition education tailored to urbanized and high-risk populations.
5. Native Amerindians and Afro-descendant health sovereignty framework: Create participatory research and health governance mechanisms that guarantee data sovereignty, benefit-sharing, and culturally adapted consent processes, ensuring genomic research and health interventions respect community rights and priorities.

Together, these policies align with a precision public health approach that links scientific evidence to national health planning, emphasizing equity, inclusion, and long-term capacity-building across Ecuador’s diverse populations.

### Future perspectives

The study of Ecuadorian populations opens multiple avenues for advancing both science and health equity.

1. Research expansion: Large-scale genomic projects should include mestizos, Afro-Ecuadorians, and diverse Indigenous groups, using genome-wide data, longitudinal cohorts, and careful integration of environmental and social determinants. This will help disentangle genetic adaptation from the effects of urbanization, poverty, and nutrition. We emphasize the need for representativeness and integration of environmental context to overcome current sampling and interpretive limitations.
2. Clinical translation: Pharmacogenomic variation in genes such as *CYP2D6*, *DPYD*, and *TPMT* should inform local treatment guidelines. Implementing targeted genetic testing in oncology, psychiatry, and pediatrics can reduce drug toxicity and improve therapeutic efficacy. We outline practical clinical applications derived from pharmacogenomic evidence.
3. Adaptation and health monitoring: High-altitude physiology, microbiome transitions, and dietary changes among Indigenous and rural communities require ongoing investigation, as they provide insights into both evolutionary biology and current risks of cardiometabolic and respiratory diseases. We highlight the importance of linking studies of evolutionary adaptation with real-time health surveillance and preventive strategies.
4. Policy and equity: Research must be community-centered, respecting genetic sovereignty and ensuring equitable access to benefits. National policies should support genetic counseling, clinical training in precision medicine, and public health programs that integrate genomic insights with interventions on nutrition, chronic disease, and environmental health. We outline explicit policy pathways that connect genomic science to national health planning and the promotion of equity.
5. Interdisciplinary collaboration: Progress will depend on cooperation across genetics, anthropology, epidemiology, and public health, with strong partnerships between local institutions and international networks. We highlight the need for sustained cross-disciplinary collaboration and capacity-building within Ecuador’s research ecosystem.

In short, Ecuador can become a regional model for how the study of underrepresented populations enriches global science while directly informing inclusive health policies. We emphasize Ecuador’s potential leadership in integrating genomic diversity with equitable public health innovation.

## Conclusion

Ecuadorian populations—mestizos, Native Amerindians, and Afro-Ecuadorians—represent a distinctive tri-hybrid genetic mosaic shaped by admixture, isolation, and environmental diversity. They display unique profiles in ancestry, immunogenetics, pharmacogenomics, and adaptation to altitude, nutrition, and disease risk. These differences demonstrate that health patterns and therapeutic responses in Ecuador cannot be extrapolated from European or Asian data alone. We emphasize the importance of interpreting genetic findings within their specific ancestral, ecological, and social contexts. Nevertheless, the current evidence remains fragmented derived from small, geographically restricted samples that seldom integrate environmental or social determinants. This underrepresentation limits both scientific insight and the applicability of precision medicine in Ecuador. We explicitly acknowledge these methodological and representational gaps, addressing concerns regarding data coverage and generalizability. Moving forward, Ecuador requires large-scale, community-engaged research that integrates genomic, environmental, and social data, alongside the systematic translation of pharmacogenomic discoveries into clinical practice and public health policy. We highlight the urgent need for well-defined translational pathways linking genomic science to equitable national health planning. Finally, the inclusion of Ecuadorian populations in international studies is both an equity imperative and a scientific opportunity to enrich global precision medicine. Ecuador is poised to assume a leading role in integrating genomic diversity with inclusive, equity-driven public health innovation.

## Abbreviations

Hb, Hemoglobin; HbAS, Sickle cell trait (heterozygous for hemoglobin S); HbSS, Sickle cell disease (homozygous for hemoglobin S); HLA, Human Leukocyte Antigen; CYP2D6, CYP2C19, DPYD, TPMT, Cytochrome P450 family enzymes and drug metabolism genes; Y-STR, Y-chromosome Short Tandem Repeat; SNP, Single Nucleotide Polymorphism; AISNP, Ancestry-Informative Single Nucleotide Polymorphism; DM2, Type 2 Diabetes Mellitus; HTN, Hypertension; PM2.5/NO_2_, Fine particulate matter and nitrogen dioxide (air pollution indicators); EHR, Electronic Health Record; ATS/ERS, American Thoracic Society/European Respiratory Society; UV, Ultraviolet; DNA, Deoxyribonucleic Acid; FAIR, Findable, Accessible, Interoperable, and Reusable (data principles); CNV, Copy Number Variation; IL-5, Interleukin 5; FeNO, Fractional exhaled Nitric Oxide; TB, Tuberculosis; SSRIs, Selective Serotonin Reuptake Inhibitors; M, Male; F, Female.
